# Strategies for Enhancing Conventional Glass Ionomer Cement—A Short Review

**DOI:** 10.3390/ma19040653

**Published:** 2026-02-08

**Authors:** Ye Zhang, Jingwei He

**Affiliations:** School of Materials Science and Engineering, South China University of Technology, Guangzhou 510641, China; yezhang1005@163.com

**Keywords:** filler enhancement, glass ionomer cement, mechanical properties, polymer modification, research progress

## Abstract

Conventional glass ionomer cement (GIC) is a reaction product formulated from glass powders and polycarboxylic acid aqueous solution. This material has garnered significant attention in restorative dentistry due to its favorable properties, including chemical adhesion to tooth structure, biocompatibility, and sustained fluoride release, coupled with its minimal pulp irritation. However, its low mechanical strength, high brittleness, and susceptibility to cracking limit its use in stress-bearing areas of teeth. To expand the clinical application scope of GIC and develop an “ideal” dental restorative material, enhancing traditional GIC is necessary. This narrative review summarizes the main strategies for enhancing GIC, covering modifications to both the powder and liquid components. The key findings indicate that incorporating reinforcing fillers into the powder or modifying the polyacid chemistry can significantly improve mechanical properties such as compressive, tensile, and flexural strength. Additionally, some modifications help maintain or enhance fluoride release. However, the translation of many laboratory-based improvements to clinical practice requires further validation. In conclusion, while numerous promising enhancement routes exist, future development should focus on synergistic approaches and rigorous clinical evaluation to advance towards high-performance, durable restorative materials.

## 1. Introduction

**Figure 1:**
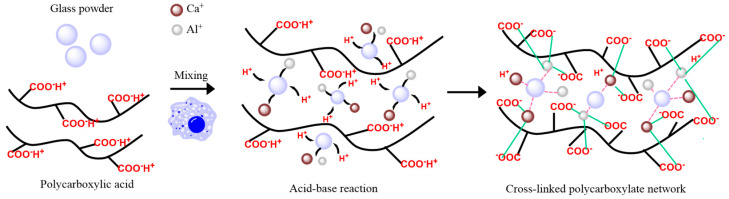
Reaction mechanism of GIC.

Glass Ionomer Cement (GIC) is fundamentally composed of powders and a liquid component. The powders consist mainly of SiO_2_, Al_2_O_3_, CaF_2_, AlF_3_, NaF, and AlPO_4_, while the liquid is primarily an aqueous solution of polycarboxylic acid, typically including acrylic acid homopolymers, acrylic acid (AA)-itaconic acid (IA) copolymers, or copolymers of AA with other unsaturated carboxylic acids [1,2,3]. Upon mixing the powders and the liquid, the carboxylate groups of the polycarboxylic acid chelate with metal ions released from the glass powders, forming a cross-linked polycarboxylate network (as shown in Figure 1) that leads to the hardening of the cement [4,5]. Once set, GIC exhibits several favorable properties [6,7,8,9], including effective adhesion to tooth structure, excellent biocompatibility, and the ability to release fluoride ions over time. These characteristics contribute significantly to its clinical utility in dentistry [9,10]. However, the application of conventional GICs, particularly in high-stress-bearing areas such as posterior restorations, is substantially constrained by several inherent limitations, most notably low mechanical strength, poor wear resistance, and high brittleness [11,12].

In response to these drawbacks, considerable research efforts have been directed toward modifying and enhancing conventional GICs to improve their mechanical performance and expand their clinical scope. These enhancement strategies can be broadly classified into two main categories: modifications to the powder’s components and modifications to the liquid components [13,14,15].

## 2. Methodology

This narrative review systematically synthesizes existing knowledge regarding enhancement strategies for glass ionomer cements, evaluates the findings and discrepancies across different studies, and identifies directions for future academic exploration. The literature search was conducted utilizing databases including Web of Science, Elsevier, SciELO, Wiley, and PubMed. Search terms comprised “glass ionomer cement,” “enhance,” “modification,” “powder,” “filler,” “liquid,” “polycarboxylic acid,” and “mechanical properties,” among others. Keywords were combined using Boolean operators (AND/OR). The search proceeded retrospectively from recent to earlier publications. Additionally, a manual backward search was performed by reviewing the reference lists of articles identified through the electronic databases.

The inclusion criteria focused on English-language original research articles and reviews concerning the enhancement and modification of conventional glass ionomer cements. Laboratory studies, review articles, and selected clinical studies were considered. The search and screening process aimed to encompass the principal enhancement strategies, including commercially successful types such as glass hybrids. However, given the narrative nature of this review and the substantial volume of research on glass ionomer cement modifications, this work does not constitute an exhaustive systematic review, and some less prominent modification approaches may not be covered.

## 3. Modification of the Powders Components

A primary strategy for enhancing GICs is the incorporation of reinforcing fillers into the powder component. Metallic particles, fibers, and inorganic fillers are among the most commonly employed [13,16]. Researchers have endeavored to improve the mechanical properties and expand the clinical applicability of GICs in dental restorations by systematically optimizing the filler characteristics, such as their type, particle size, morphology, and incorporation ratio.

### 3.1. Metallic Materials

Numerous studies [17,18,19] have investigated metal-reinforced glass ionomer cements, utilizing metals such as Au, Ag, Sn, and Ti, as well as certain metal alloys. The primary mechanism of metal-reinforced GIC involves the physical interlocking of metal particles within the polycarboxylate cross-linked network, which enhances mechanical properties by dispersing stress [18,20]. As early as the 1980s, researchers introduced metallic silver into GICs to improve their mechanical strength [21]. Research by Walls et al. [22] has demonstrated that the addition of sintered silver particles to glass ionomer cement can significantly enhance its compressive strength and fatigue resistance. Kerby et al. [23] reported that the GIC prepared by mixing acid-treated stainless-steel powder with glass-ionomer powders exhibited a significant increase in compressive and tensile strength compared to commercially available silver-reinforced GIC. Subsequently, Chung [20] compared the reinforcing effects of silver and high-copper amalgam powders on GICs. The results demonstrated that both additives enhanced the tensile strength and hardness of conventional GICs, with a 40% proportion of high-copper amalgam showing a more significant reinforcing effect. However, the study also indicated that the fluoride release of these metal-reinforced GICs was lower than that of their conventional counterparts [20,24]. This reduction in fluoride release has become a recognized drawback of nearly all metal-reinforcement approaches, coupled with their generally inferior adhesion to tooth structure. Nevertheless, recent studies have also indicated that silver nanoparticles (AgNPs) at specific concentrations (e.g., 1–2.5%) can enhance fluoride ion release, while metal additives such as zinc, strontium, titanium, and zirconium in the form of oxides demonstrate even greater effectiveness in promoting fluoride ion release at certain concentrations [25].

### 3.2. Metal Oxides

Building on the metal reinforcement research, subsequent efforts have shifted towards the incorporation of metal oxides. After oxidation treatment, the AgSnZnAl alloy used by Sarkar [26] was found to provide better reinforcement than commercially available metal-reinforced GIC. Tests revealed that the metal oxides on the alloy surface chemically reacted with polycarboxylic acid, indicating that the reinforcement mechanism involves chemical bonding [27,28]. Extensive studies have utilized nano-sized metal oxides, such as ceria (CeO_2_), zirconia (ZrO_2_), magnesia (MgO), and zinc oxide (ZnO), which enhance the GIC by filling the interstitial spaces between the glass particles [29]. The reinforcement mechanism of nano-metal oxides primarily involves filling the gaps between glass particles, reducing pores and microcracks, and thereby enhancing the material’s density and bonding strength [29,30,31]. Gu et al. [32] replaced the silver amalgam in metal-reinforced GICs with yttria-stabilized zirconia (YSZ). Their findings demonstrated a significant improvement in the tensile strength of the modified cement. In another study, Jairam et al. [33] incorporated CeO_2_ nanoparticles into the glass powder. The results indicated that at an optimal concentration of 8%, the microhardness, compressive strength, and tensile strength increased by 26.6%, 35.5%, and 50%, respectively.

Beyond single-component modifications, the synergistic effect of incorporating two or more metal oxides has also been explored. For example, Monmaturapoj et al. [34] investigated the reinforcement of GICs using a combination of MgO and BaO. While metal oxides can improve certain mechanical properties of GICs, they also introduce drawbacks such as prolonged setting time, reduced adhesive strength, and poor color matching [29]. However, compared to metallic fillers, metal oxides are more conducive to maintaining or even enhancing the sustained fluoride release capability of GICs, thereby addressing a key limitation associated with traditional metal reinforcement.

### 3.3. Fiber

Early GICs were initially adopted as luting agents in dentistry, but their limited mechanical properties prevented widespread use in restorative applications. Consequently, researchers have begun to explore the reinforcement of GIC with fibers. The reinforcement mechanism of fibers lies primarily in their anisotropic properties, which effectively inhibit crack propagation. Additionally, the composition and surface characteristics of different fibers contribute to interfacial bonding [35,36,37]. A significant advancement was made by Kobayashi et al. [38], who pioneered the use of glass fibers (CPSA) as reinforcing agents. They found that a glass fiber mass ratio of 60% yielded the maximum tensile and flexural strengths, which were 1.8 and 4.5 times higher than those of conventional GICs, demonstrating the high efficacy of glass fiber reinforcement. It should be noted, however, that incorporating CPSA glass fibers can significantly prolong the setting time of GIC. Moreover, not all glass fibers are suitable for this purpose; they must possess a specific chemical composition that is compatible with the GIC system and capable of participating in the setting reaction. Based on the work with glass fibers, Bao et al. [39] investigated silane-treated basalt fibers for GIC reinforcement. Their research confirmed that basalt fibers significantly enhance the mechanical properties of GICs, with an optimal performance achieved using 7 wt.% of 2 mm long fibers.

In addition to inorganic fibers, researchers have also explored natural fibers. Numerous studies have investigated the reinforcing effect of cellulose fibers on GIC. Findings demonstrate that incorporating these fibers at specific proportions improves its compressive strength [40,41] and fracture toughness [42]. In particular, nano-scale cellulose fibers [43] have been shown to provide a comprehensive improvement in the mechanical properties of GIC. Abou Neel et al. [36] and Kuscu et al. [37] utilized flax and hemp fibers to reinforce GICs. These natural fibers not only improve the mechanical properties to a certain extent but also offer the potential for more environmentally sustainable and sustainable development. However, the incorporation of natural fibers typically increases the viscosity of GIC and shortens its working time, which can make clinical mixing and shaping more difficult. Concurrently, whiskers [44,45,46] have emerged as another form of reinforcement for modifying and enhancing glass ionomer cements.

### 3.4. Hydroxyapatite

Hydroxyapatite (HA), the primary inorganic constituent of human bones and teeth, has been incorporated into GICs in an attempt to combine biocompatibility with mechanical enhancement [47,48]. In addition to physical reinforcement, its enhancement mechanism also involves HA participating in acid-base reactions. During this process, phosphate and calcium ions react with the inorganic/organic components within the GIC network. When H^+^ ions are released and attack the HA, more Ca^2+^ ions become available for cement formation, polymeric salt bridge formation, and cross-linking, thereby strengthening the GIC [49,50]. Lucas et al. [51] added 8 wt.% HA to the powder of a conventional GIC. The mixture was manually blended with the liquid component, an aqueous solution of polyacrylic and itaconic acids, at a powder-to-liquid ratio of 3:6. Their results indicated that the fracture strength of this modified cement reached a substantially high level within the first 15 min, whereas the conventional GIC required 24 h of curing to achieve its maximum fracture strength. Subsequent studies have also reported that the incorporation of HA can enhance the flexural strength, diametral tensile strength and toughness of GICs [52,53,54]. In recent years, researchers have begun to utilize hydroxyapatite nanoparticles for reinforcement [55,56,57].

However, the advantage of HA lies less in its mechanical reinforcement and more in its superior biocompatibility and ion-releasing properties. This has established HA modification as a mainstream research direction. Consequently, current research often focuses on combining HA with one or more additional materials [58,59,60,61] to achieve synergistic enhancement of GICs.

### 3.5. Glass Hybrids

The modification and enhancement of GIC by using glass hybrid systems no longer relies on the chemically less reactive fluoroaluminosilicate glass powder found in traditional GICs. Instead, a specially designed, highly reactive glass filler is employed. The key feature of this approach is the use of strontium and lanthanum-containing aluminosilicate glass to partially replace calcium aluminosilicate glass. These elements not only enhance the stability of the glass but also release beneficial ions during the reaction. Through special processing, the glass particles are finer with a larger specific surface area, allowing for more thorough and rapid reactions with polycarboxylic acid [62,63]. Meanwhile, the glass hybrid system enables extremely high filler content.

An important and commercially successful improvement is the development of glass hybrid or glass hybrid restorative materials. Such materials represent an advanced evolution of traditional GICs. Products such as ChemFil Rock and EQUIA Forte Fil fall into this category. Many researchers have investigated the mechanical properties of these glass ionomer dental restorative materials. The enhancement of ChemFil Rock is achieved through the combined use of highly reactive glass fillers and optimized polymers [62]. It exhibits higher compressive strength and diametral tensile strength compared to traditional GICs [64]. Although it performs well, it has notable shortcomings, such as insufficient hardness [63], and is marketed for restorations in non-load-bearing areas [65]. EQUIA Forte Fil’s glass hybrid innovation is achieved through the introduction of ultrafine, highly reactive glass particles, dispersed within the conventional glass ionomer structure. With the addition of a higher molecular weight polyacrylic acid, the new glass hybrid formulation builds a high strength restorative [62,64,65]. EQUIA Forte Fil outperforms traditional GICs in terms of flexural strength and surface hardness, while its compressive strength and diametral tensile strength are slightly higher than those of Fuji IX [65,66]. Its successful application in load-bearing restorations [61] is precisely attributed to this optimization strategy for the powder formulation, which simultaneously addresses several limitations of traditional GICs. Although EQUIA Forte Fil is superior to conventional GICs, its fracture resistance may be insufficient when subjected to occlusal forces [65].

### 3.6. Other Materials

In addition to the aforementioned material reinforcement, studies have also utilized chitosan and its derivatives. For instance, Kashyap et al. [67] incorporated carboxymethyl chitosan (CMC) with glass powder. CMC, which possesses multiple functional groups such as carboxyl, hydroxyl, and amino groups, can form stronger ionic and hydrogen bonds with ions in the GIC. This enhances the interfacial bonding and improves the mechanical properties.

Furthermore, nanocarbon materials like graphene [68,69,70,71] and carbon nanotubes [72,73,74,75,76] have shown emerging potential in GIC reinforcement, owing to their exceptionally high specific strength and unique properties. Benevides et al. [70] reinforced GIC using reduced graphene oxide and its composite materials. Their results indicated that while the improvements in compressive and tensile strength were moderate, the enhancement in hardness was significant. While Sun et al. [71] employed fluorinated graphene, which effectively improved the compressive strength and hardness, the flexural strength across all experimental groups showed no statistically significant differences compared to the control. Additionally, Goyal et al. [76] found that the addition of 1% multi-walled carbon nanotubes yielded the optimal mechanical strength for GIC. However, the dark color of the resulting composite precludes its use in anterior teeth or visible restorations, significantly limiting its clinical applicability and effective dosage range.

The ongoing advancement of science and technology is continually introducing a growing variety of materials, particularly at the nanoscale [5,77,78], for the enhancement of glass ionomer cement. Overall, the enhancement effects of different materials are summarized in Table 1.

## 4. Modification of the Liquid Component

As the concentration of hydrogen ions increases in the polyacrylic acid solution of conventional glass ionomer cement, the ionization of the remaining carboxyl groups is progressively suppressed. This, in turn, prevents these carboxyl groups from fully participating in cross-linking reactions with the metal ions leached from the glass powder. As a result, the cross-linking density is compromised, which adversely affects the mechanical properties of the cement. To address this, the polycarboxylic acid is commonly modified by copolymerizing it with a third monomer featuring longer side-chain carboxyl groups. These introduce steric hindrances that disrupt intermolecular hydrogen bonds, thereby enhancing carboxyl group reactivity during setting [14,79]. Simultaneously, some studies have enhanced GIC by directly incorporating materials into the liquid component. These materials primarily form a reinforcing network within the GIC matrix through hydrogen bonding, ionic bonding, or physical cross-linking, thereby improving stress distribution and interfacial bonding [79].

### 4.1. Liquid-Phase Additives

In recent years, researchers have introduced natural or modified biomaterials into the liquid component of GIC to enhance its overall performance. Chang et al. [80] reported that adding 0.01 wt.% collagen to the liquid significantly increased the compressive strength (from 172.0 MPa to 204.2 MPa). Menezes-Silva et al. [81] incorporated cellulose nanocrystals (0.2 wt.% of the total mass) into the polyacrylic acid liquid component of GIC, followed by ultrasonic dispersion before mixing with the powder; the results showed a notable improvement in both compressive strength and diametral tensile strength. Rini et al. [82] and Al-Mofty et al. [83] enhanced GIC using cellulose derived from rice husks, processed via acid hydrolysis and one-step sodium hypochlorite treatment to obtain nanocrystalline cellulose and micro-scale cellulose, respectively. Both studies demonstrated that rice husk cellulose effectively improved the mechanical properties of GIC, particularly in terms of hardness and bond strength. Furthermore, Petri et al. [84] and Bao et al. [85] found that very low concentrations of chitosan (0.0044 wt.%) or carboxymethyl chitosan (0.0125 wt.%), respectively, could significantly enhance flexural strength and fluoride ion release. Chitosan forms a network structure with GIC components through hydrogen bonding and electrostatic interactions, reducing interfacial tension and improving mechanical properties. While the addition of trace amounts of biomaterials to the liquid component of GIC can effectively enhance its mechanical strength, biocompatibility, and functional properties, it should be noted that the improvement in mechanical performance is selective rather than comprehensive. Furthermore, this approach may introduce new structural defects or instability.

### 4.2. Synthesis of Novel Copolymers

#### 4.2.1. Amino Acid Derivatives

Kao et al. [86] synthesized two amino acid-derived monomers, N-acryloyl glutamic acid (AGA) and N-acryloyl-6-aminohexanoic acid (AACA), which were subsequently copolymerized with AA and IA to form polyacids with varying compositions. The GICs prepared from these modified polyacids exhibited improved mechanical properties. Expanding on this approach, Wu et al. [87] prepared methacrylate or acrylate derivatives from six natural amino acids and fabricated the corresponding terpolymers. Their research indicated that all amino acid-modified GICs exhibited significantly enhanced compressive and flexural strengths. In a notable study, Moshaverinia et al. [88] synthesized a proline derivative and its corresponding terpolymer with AA and IA to modify a commercial Fuji II GIC. The results confirmed substantial property enhancements, with increases of 27% in compressive strength, 170% in biaxial flexural strength, and 94% in diametral tensile strength compared to the unmodified cement.

**Figure 2:**
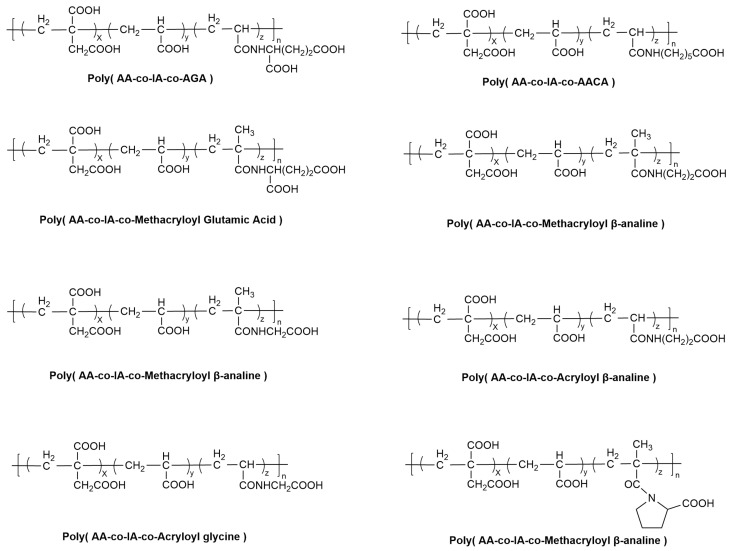
Structures of Poly (AA-co-IA-co-Amino acid derivatives).

In summary, amino acid modification of GICs introduces flexible spacer groups (as shown in Figure 2) that effectively reduce the steric hindrance between the carboxyl groups and the polymer backbone. This enhances their chelation ability with Ca^2+^ and Al^3+^ ions from the glass powder, leading to the observed significant enhancement in the mechanical performance of the material. However, as the molecular weight of the polyacid increases, the viscosity of the cement rises, making mixing and placement more challenging. Concurrently, the working time is significantly reduced, potentially compromising the clinically manageable working window.

#### 4.2.2. N-Vinyl Lactam

Yamazaki et al. [89] copolymerized varying contents of N-vinylpyrrolidone (NVP) with AA and maleic acid (MA). They determined that the terpolymer with an AA-MA-NVP ratio of 8:1:1 yielded GICs with optimal mechanical properties, which were superior or comparable to those of the commercial Fuji IX.

Xie et al. [90] found that a terpolymer with an AA-IA-NVP ratio of 7:1:3 resulted in a GIC with significantly higher flexural strength. Their study also established a positive correlation between the molecular weight of the terpolymer and the flexural strength of the resulting cement. Furthermore, the microhardness of this modified GIC was superior or comparable to that of most commercial products [91]. It was also noted that the compatibility between the NVP-modified copolymer and commercial glass powders was suboptimal. The development of dedicated glass powders is therefore necessary to achieve optimal performance.

**Figure 3:**
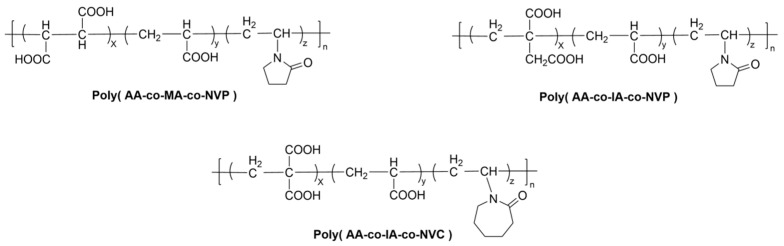
Structures of Poly (AA-co-IA/MA-co-N-vinyl lactam).

Building on this foundation, Moshaverinia et al. [92,93] synthesized an AA-IA-NVC (8:1:1) terpolymer. Testing revealed improvements in the flexural strength, compressive strength, tensile strength and fracture toughness of the modified cement. Both NVP and NVC function as spacer groups (as shown in Figure 3) that reduce steric hindrance, thereby promoting the formation of a greater number of salt bridges within the set matrix and ultimately enhancing the material’s properties.

#### 4.2.3. Alkyl-Enoic Acids

**Figure 4:**
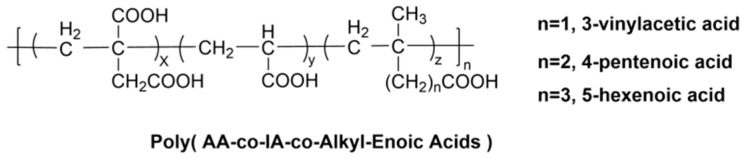
Structures of Poly (AA-co-IA-co-Alkyl-Enoic acids).

The insufficient mechanical strength of conventional GICs restricts their use in load-bearing applications. Studies show that incorporating flexible alkenoic acid spacers into the polycarboxylic acid backbone (as shown in Figure 4) effectively addresses this limitation. Liang et al. [94,95,96] systematically demonstrated that introducing spacers such as 3-butenoic acid, 4-pentenoic acid, and 5-hexenoic acid into the polyacid structure significantly enhances GIC mechanical properties. Their research optimized key parameters including spacer content, polyacid molecular weight, and concentration. A critical finding was the identification of 12 mol% as the optimal addition level. Modification with 3-butenoic acid proved particularly effective, yielding a flexural strength of 52.29 MPa (a 23.1% increase) and a flexural modulus of 16.30 GPa, indicating superior rigidity and load-bearing capacity while preserving structural integrity.

Despite significant improvements in mechanical properties, its suitability for high-stress-bearing areas, such as posterior restorations, requires further validation through comprehensive comparative studies with established materials like ceramics and resin composites. Simultaneously, the alkyl-enoic acid modified GICs maintained low water sorption and sustained fluoride release capability, demonstrating promising clinical potential as high-strength, long-term anticariogenic restorative materials. As summarized in Table 2, the different liquid agents varied in their enhancement effects. 

## 5. Conclusions and Future Perspectives

While conventional GICs exhibit relatively poor mechanical strength, numerous studies have been conducted to enhance their properties. However, different reinforcement methods each have their own advantages and limitations, as summarized in Table 3. In addition to direct modifications of the powder and liquid components, the mechanical performance of GICs is also influenced by various factors, such as the particle size [97], shape [98], and surface condition of the powder, the molecular weight [99] and concentration [100] of the liquid, and the powder-to-liquid ratio [101]. Consequently, a more systematic investigation into these influencing factors is warranted.

As shown in Table 3, most enhancement strategies require balancing the strength, performance, and handling properties of glass ionomer cement. Improving mechanical properties often comes at the cost of sacrificing other clinically essential characteristics, such as reduced working time, increased viscosity affecting clinical handling, compromised esthetics, altered fluoride release properties, or inconsistent adhesion to tooth structures. While the reviewed studies demonstrate promising laboratory outcomes for enhanced GICs, several challenges and limitations must be acknowledged when considering clinical translation. First, there is significant heterogeneity among studies in terms of materials, modification methods, filler concentrations, and testing protocols, making direct comparisons difficult. Second, most evidence comes from in vitro studies, which may not fully replicate the complex oral environment, including fatigue loading, thermal cycling, and biofilm challenge. The long-term clinical performance, biocompatibility, and wear characteristics of many novel modifications remain to be validated through rigorous clinical trials.

Future research must move beyond the single metric of “strength” and focus on a comprehensive multi-dimensional performance evaluation. Current research is trending towards diversified and composite strategies. The future lies in ‘synergistic enhancement’—a hybrid approach combining powder and liquid modifications or employing multi-functional fillers to develop GICs that simultaneously offer superior mechanical strength, biocompatibility, and antibacterial efficacy.

Additionally, future research should also focus on the perspectives to bridge the gap between laboratory innovation and clinical application. Primarily, to facilitate the clinical translation of these advanced materials, it is imperative to establish comprehensive performance standards and conduct long-term, large-scale clinical studies to validate their long-term performance in demanding applications, such as stress-bearing restorations. Subsequently, well-designed, long-term clinical studies are imperative to confirm the durability, safety, and efficacy of these advanced materials in vivo.

## Figures and Tables

**Table 1:** Summary of the enhancement effects of different materials with the powder’s components.

Material Types	Specific Materials		Optimal Content	Enhancement Effect
Metallic materials	Sintered silver particles (Ketac-Silver) [22]		Commercial Product	1. Compressive strength: 216.9 MPa (vs. Ketac-Fil: 182.0 MPa) 2. Compressive fatigue limit: 96.52 MPa (vs. Ketac-Fil: 69.60 MPa) 3. Flexural strength: 28.94 MPa (lower than that of Ketac-Fil, 44.63 MPa)
	Stainless-steel powder [23]		Filler/glass = 0.5/1 (g/g), 17 vol%	1. Compressive strength: 268.3 MPa (vs. Ketac-Silver: 175.3 MPa and Miracle Mix: 168.7 MPa) 2. Radial tensile strength: 22.7 MPa (vs. Ketac-Silver: 14.2 MPa and Miracle Mix: 10.9 MPa)
	High-copper amalgam alloy (FAL) [20]		72 wt.%	1. Diametral tensile strength: 15.1 MPa (vs. Fuji II: 9.2 MPa) 2. Barcol hardness: 65.8 BHN (vs. Fuji II: 20.1 BHN)
Metal oxides	Zirconia (YSZ) [32]		Volumetric replacement of amalgam	1. Diametral tensile strength: 10.58 MPa/1 d→11.21 MPa/1 m (vs. Miracle Mix: 6.31→7.32 MPa, and Fuji IX: 11.87→13.07 MPa) 2. Compressive strength: 150.82 MPa/1 d→177.28 MPa/1 m (no significant difference from Miracle Mix or Fuji IX)
	Cerium oxide nanoparticles [33]		8 wt.%	1. Vickers hardness: 40.90 VH (26.6% increase vs. conventional GIC, 30.56 VH) 2. Compressive strength: 244.9 MPa (35.5% increase vs. conventional GIC, 177.2 MPa) 3. Tensile strength: 33.46 MPa (50% increase vs. conventional GIC, 20.68 MPa)
	BaO and MgO [34]		Equal amounts of BaO/MgO	Compressive strength: ~110 MPa (significantly increased vs. original glass without BaO/MgO)
Fiber	Glass short fibers (CPSA) [38]		60 wt.% (aspect ratio 5.0, Ø9.7 µm)	1. Flexural strength: 35.0 MPa (vs. control, 7.8 MPa) 2. Diametral tensile strength: 17.9 MPa (vs. control, 10.8 MPa)
	Basalt Fiber [39]		7 wt.% (length 2 mm, Ø13.3 µm)	1. Flexural strength: 43.93 ± 6.20 MPa (vs. Fuji IX: 27.72 ± 3.01 MPa) 2. Compressive strength: 142.57 ± 20.86 MPa (vs. Fuji IX: 94.71 ± 28.03 MPa) 3. Fracture toughness: 1.24 ± 0.32 MPa·m^1/2^ (vs. Fuji IX: 0.38 ± 0.07 M·Pam^1/2^) 4. Water sorption: 7.34 ± 0.41% (vs. Fuji IX: 5.76 ± 0.31%)
	Cellulose fibers	1. Obtained from eucalyptus wood [40]	7.24 wt.%	1. Compressive strength: 62.57 ± 18.10 MPa (vs. control, 49.15 ± 46.00 MPa) 2. Wear resistance (mass loss): 0.053 ± 0.012 g (vs. control, 0.081 ± 0.002 g) 3. Bond strength: 0.055 ± 0.948 MPa (vs. control, 0.008 ± 0.236 MPa)
		2. Mechanically processed cellulosic fibers [41]	7.24 wt.%	1. Compressive strength: 47.17 ± 8.98 MPa (vs. G1-GIC, 23.66 ± 8.48 MPa) 2. Diametral tensile strength: 49.56 ± 5.03 MPa (vs. G1-GIC, 22.37 ± 6.04 MPa)
Fiber	Cellulose fibers	3. Cellulose microfibers [42]	For compressive strength: 1 wt.% For fracture toughness: 5 wt.%	1. Compressive strength: 116 MPa (vs. Fuji IX: ~100–110 MPa) 2. Fracture toughness, K_max (5%): 0.9 M·Pam^1/2^ (vs. Fuji IX: 0.4 M·Pam^1/2^) 3. Work of fracture: increased progressively with fiber content
		4. Cellulose nanofibers [43]	For flexural/compressive: 4 wt.% For tensile: 6 wt.%	1. Flexural strength (4%): 24.13 ± 2.07 MPa (vs. control, 16.36 ± 3.12 MPa) 2. Compressive strength (4%): 119.59 ± 7.54 MPa (vs. control, 102.26 ± 9.40 MPa) 3. Diametral tensile strength (6%): 13.59 ± 0.72 MPa (vs. control, 10.61 ± 1.39 MPa)
	Flax fiber [36]		For compressive strength: 25 wt.% For flexural strength/ductility: 5 wt.% (length ~675 µm)	1. Compressive strength(25%): 250 ± 50 MPa (vs. conventional GIC: 148 ± 12 MPa) 2. Flexural strength (5%): 42 MPa (vs. conventional GIC: 20 ± 2 MPa)
	Hemp fiber [37]		1 wt.%	1. Flexural strength: 0.2801 MPa (vs. conventional GIC: 0.1660 MPa) 2. Surface roughness: 0.486 µm (vs. conventional GIC: 1.222 µm)
Hydroxyapatite (HA)	HA Whiskers [52]		19 wt.% (P/L = 1.75)	Flexural strength: 23.8 ± 2.0 MPa (vs. Fuji IX GP: 9.0 ± 2.3 MPa, *p* < 0.001)
	Experimental HA [53]		10 wt.% (15 μm)	Diametral tensile strength: 6.74 ± 1.55 MPa (vs. Fuji II: 4.12 ± 0.67 MPa, *p* ≤ 0.005)
	Commercial HA [53]		5 wt.% (<1 μm)	Diametral tensile strength: 6.98 ± 2.26 MPa (vs. Fuji II: 4.12 ± 0.67 MPa, *p* ≤ 0.005)
	HA granules [52,54]		8 wt.% (P/L = 3.6)	1. Fracture toughness (15 min): 0.56 ± 0.10 MPa·m^1/2^ (vs. Fuji IX GP: 0.36 ± 0.06) (24 h): 0.58 ± 0.09 MPa·m^1/2^ (vs. Fuji IX GP: 0.45 ± 0.06) 2. Flexural strength: 25.8 ± 1.6 MPa (vs. Fuji IX GP: 18.0 ± 3.8 MPa, *p* < 0.01)
Glass hybrids	ChemFil Rock [64]		Commercial Product	1. Compressive strength: 171.3 ± 30.99 MPa (vs. Fuji IX: 131.2 ± 10.03) 2. Diametral tensile strength: 19.1 ± 3.44 MPa (vs. Fuji IX: 14.1 ± 2.13)
	EQUIA Forte Fil [66]		Commercial Product	1. Compressive strength: 112.48 MPa (vs. Fuji IX: 108.59) 2. Diametral tensile strength: 12.43 MPa (vs. Fuji IX: 10.09)
Other materials	Carboxymethyl chitosan [67]		10 wt.%	1. Compressive strength: 157.45 MPa (vs. commercial GIC: 149.64 MPa) 2. Flexural strength: 18.76 MPa (vs. commercial GIC: 12.62 MPa)
	Reduced graphene oxide [70]		0.1 wt.%	Knoop hardness: 41.15 ± 1.14 KHN (vs. control: 38.47 ± 2.44 KHN)
	Fluorinated graphene [71]		2 wt.%	1. Compressive strength: 181.2 ± 9.4 MPa (vs. control: 113.6 ± 4.8 MPa) 2. Vickers hardness: 69.4 ± 1.9 kg/mm^2^ (vs. control: 43.2 ± 2.4 kg/mm^2^)
	Multi-walled carbon nanotubes [76]		1 wt.%	Compressive strength: 51.7 ± 0.8 MPa (vs. control: 45.0 ± 1.2 MPa)

**Table 2:** Summary of the enhancement effects of different liquid components.

Strategies	Liquid Types	Specific Materials	Optimal Content	Enhancement Effect
Additives		Collagen [80]	0.01% (*w*/*w*)	Compressive strength: 204.2 ± 32.9 MPa (vs. Fuji-II: 172.0 ± 22.9 MPa)
	Cellulose	Cellulose nanocrystals [81]	0.2 wt.%	1. Compressive strength: 130.28 ± 25.30 MPa (vs. Ma: 98.42 ± 3.67 MPa) 2. Diametral tensile strength: 54.13 ± 19.22 MPa (vs. V: 30.16 ± 12.68 MPa)
		Nanocrystalline cellulose from rice husks [82]	1% (*w*/*w*)	1. Vickers hardness: 90.04 ± 1.89 VHN (vs. control: 70.50 ± 1.29 VHN) 2. Shear bond strength: 8.00 ± 0.17 MPa (vs. control: 5.33 ± 0.31 MPa)
		Micro-scale cellulose from rice husks [83]	3% (*w*/*w*)	Compressive strength: +130% (vs. control)
	Chitosan and derivatives	Chitosan [84]	0.0044 wt.%	Flexural strength: 18.14 ± 3.26 MPa (vs. GIR-Control: 14.27 ± 2.60 MPa)
		Carboxymethyl chitosan [85]	0.0125 wt.%	1. Flexural strength: 34.45 ± 2.51 MPa (vs. C-GIC: 27.72 ± 3.01 MPa) 2. Fracture toughness: 0.51 ± 0.07 MPa·m^1/2^ (vs. C-GIC: 0.38 ± 0.07 MPa·m^1/2^)
Novel copolymers	Amino acid derivatives	N-acryloyl glutamic acid (AGA) [86]	Poly(AA ^1^-IA ^2^-AGA) (10:1:1)	1. Diametral tensile strength: 22.2 ± 1.6 MPa (vs. Fuji II: 13.4 ± 1.5 MPa) 2. Compressive strength: 195.0 ± 11.4 MPa (vs. Fuji II: 168.2 ± 11.0 MPa) 3. Flexural strength: 38.0 ± 4.4 MPa (vs. Fuji II: 8.8 ± 2.03 MPa)
			Poly(AA-IA-AGA) (10:1:4)	1. Diametral tensile strength: 20.1 ± 3.3 MPa (vs. Fuji II: 13.4 ± 1.5 MPa) 2. Compressive strength: 212.0 ± 9.2 MPa (vs. Fuji II: 168.2 ± 11.0 MPa) 3. Flexural strength: 34.1 ± 3.3 MPa (vs. Fuji II: 8.8 ± 2.03 MPa)
		N-acryloyl-6-aminocaproic acid (AACA) [86]	Poly(AA-IA-AACA) (6:1:1)	1. Diametral tensile strength: 17.5 ± 3.5 MPa (vs. Fuji II: 13.4 ± 1.5 MPa) 2. Compressive strength: 180.0 ± 9.8 MPa (vs. Fuji II: 168.2 ± 11.0 MPa) 3. Flexural strength: 28.0 ± 4.6 MPa (vs. Fuji II: 8.8 ± 2.03 MPa)
			Poly(AA-IA-AACA) (10:1:1)	1. Diametral tensile strength: 19.6 ± 2.8 MPa (vs. Fuji II: 13.4 ± 1.5 MPa) 2. Compressive strength: 183.0 ± 10.7 MPa (vs. Fuji II: 168.2 ± 11.0 MPa) 3. Flexural strength: 31.0 ± 3.1 MPa (vs. Fuji II: 8.8 ± 2.03 MPa)
		Methacryloyl glutamic acid (MGA) [87]	Poly(AA-IA-MGA) (8:2:1)	1. Flexural strength: 55.14 ± 6.47 MPa (vs. Fuji IX: 15.79 ± 0.67 MPa) 2. Compressive strength: 206.0 ± 21.7 MPa (vs. Fuji II: 191.1 ± 19.1 MPa)
		Methacryloyl β-alanine (MBA) [87]	Poly(AA-IA-MBA) (8:2:1)	1. Flexural strength: 62.96 ± 2.92 MPa (vs. Fuji IX: 15.79 ± 0.67 MPa) 2. Compressive strength: 221.5 ± 27.3 MPa (vs. Fuji II: 191.1 ± 19.1 MPa)
		Methacryloyl glycine (MG) [87]	Poly(AA-IA-MG) (8:2:1)	1. Flexural strength: 60.80 ± 6.51 MPa (vs. Fuji IX: 15.79 ± 0.67 MPa) 2. Compressive strength: 228.2 ± 13.3 MPa (vs. Fuji II: 191.1 ± 19.1 MPa)
		AGA [87]	Poly(AA-IA-AGA) (8:2:1)	1. Flexural strength: 70.83 ± 1.54 MPa (vs. Fuji IX: 15.79 ± 0.67 MPa) 2. Compressive strength: 235.0 ± 11.1 MPa (vs. Fuji II: 191.1 ± 19.1 MPa)
Novel copolymers	Amino acid derivatives	Acryloyl β-alanine (ABA) [87]	Poly(AA-IA-ABA) (8:2:1)	1. Flexural strength: 70.83 ± 1.54 MPa (vs. Fuji IX: 15.79 ± 0.67 MPa) 2. Compressive strength: 235.0 ± 11.1 MPa (vs. Fuji II: 191.1 ± 19.1 MPa)
		Acryloyl glycine (AG) [87]	Poly(AA-IA-AG) (8:2:1)	1. Flexural strength: 64.10 ± 6.07 MPa (vs. Fuji IX: 15.79 ± 0.67 MPa) 2. Compressive strength: 193.2 ± 14.9 MPa (vs. Fuji II: 191.1 ± 19.1 MPa)
		Methacryloyl proline (MP) [88]	Poly(AA-IA-MP) (8:2:1)	1. Diametral tensile strength: 26.2 ± 11.2 MPa (vs. Fuji II: 13.5 ± 12.3 MPa) 2. Compressive strength: 210.2 ± 9.9 MPa (vs. Fuji II: 166.5 ± 16.2 MPa) 3. Biaxial flexural strength: 46.9 ± 4.5 MPa (vs. Fuji II: 17.2 ± 4.7 MPa)
	N-vinyl lactam	N-vinylpyrrolidone (NVP) [89]	Poly(AA-MA ^3^-NVP) (8:1:1)	1. Flexural strength: 46.0 ± 7.07 MPa (vs. Fuji IX: 32.1 ± 11.2 MPa) 2. Diametral tensile strength: 21.6 ± 3.07 MPa (vs. Fuji IX: 20.5 ± 2.53 MPa) 3. Compressive strength: 277 ± 31.0 MPa (vs. Fuji IX: 273 ± 16.1 MPa)
		NVP [90,91]	For flexural strength: Poly(AA-IA-NVP) (7:1:3) For knoop hardness: Poly(AA-IA-NVP) (7:3:1)	1. Flexural strength: 31.40 ± 2.705 MPa (85% increase vs. Ketac-Molar) 2. Knoop hardness: comparable to Fuji II and superior to a-Silver
		N-vinyl caprolactam (NVC) [92,93]	Poly(AA-MA-NVC) (8:1:1)	1. Compressive strength: 303 ± 32.8 MPa (vs. Fuji IX: 236 ± 41.5 MPa) 2. Diametral tensile strength: 38.3 ± 10.9 MPa (vs. Fuji IX: 19.6 ± 11.4 MPa) 3. Biaxial flexural strength: 82.2 ± 12.8 MPa (vs. Fuji IX: 41.3 ± 10.5 MPa) 4. Fracture toughness, K_IC: 0.58 ± 0.09 MPa·m^1/2^ (vs. Fuji IX: 0.43 ± 0.08 MPa·m^1/2^)
	Alkyl-Enoic acids	3-butenoic acid (VA) [94,96]	8 mol%	1. Flexural modulus: 19.00 ± 1.06 GPa (vs. Fuji IX: ~14.0 GPa) 2. Compressive strength: 221.35 ± 17.06 MPa (vs. Fuji IX: ~120 MPa) 3. Flexural strength: 54.14 ± 5.29 MPa (vs. Fuji IX: ~29.8–31.2 MPa)
			12 mol%	Flexural strength: 52.29 ± 4.75 MPa (vs. PCA-0: 42.49 ± 1.68 MPa)
		4-pentenoic acid (PA) [95,96]	5 wt.%	1. Compressive strength: 36.7 ± 5.9 MPa (vs. Fuji IX: 29.8 ± 3.9 MPa) 2. Flexural strength: 34.1 ± 3.3 MPa (vs. Fuji II: 8.8 ± 2.03 MPa)
			12 mol%	Flexural strength: 47.85 ± 3.88 MPa (vs. PCA-0: 42.49 ± 1.68 MPa)
		5-hexenoic acid (HA) [96]	12 mol%	Flexural strength: 41.84 ± 3.23 MPa (vs. PCA-0: 42.49 ± 1.68 MPa)

^1^ AA: Acrylic acid, ^2^ IA: Itaconic acid, ^3^ MA: Maleic acid.

**Table 3:** Summary of advantages and disadvantages of different strategies of enhancement.

Strategies	Material Types	Specific Materials	Advantages	Disadvantages
Powders	Metallic materials	Sintered silver particles (Ketac-Silver) [22]	1. Significantly increases compressive strength and fatigue limit 2. High resistance to acid erosion 3. Commercially mature product	1. Reduced flexural strength and modulus of elasticity, making the material more brittle 2. Metallic gray color, poor esthetics 3. Bond strength to dentin may be relatively low
		Stainless-steel powder [23]	1. Exceptionally high compressive and tensile strength, surpassing contemporary commercial products 2. Good acid erosion resistance 3. Strong ionic bonding at the interface can be achieved through surface treatment	1. Metallic color, unaesthetic 2. Requires complex acid pretreatment of the powder, increasing preparation difficulty
		High-copper amalgam alloy (FAL) [20]	1. Significantly improves hardness and tensile strengthwith simple preparation 2. High-copper amalgam (FAL) performs best, showing substantial property enhancement	1. Significantly reduces fluoride release, potentially weakening the anticaries advantage 2. Silver-gray color, poor esthetics 3. Weak interfacial bonding between metal and matrix, affecting long-term durability
	Metal oxides	Zirconia (YSZ) [32]	1. Significantly improves diametral tensile strength, superior to silver-amalgam reinforced products 2. Tooth-colored, providing excellent esthetics 3. Good interfacial bonding, with mechanical properties improving over time	1. No significant improvement in compressive strength, comparable to conventional GIC 2. As a bioceramic, the cost may be higher than that of conventional glass powders
		Cerium oxide nanoparticles [33]	1. Comprehensively enhances mechanical properties (hardness +26.6%, compressive strength +35.5%, tensile strength +50%) 2. Imparts potent antibacterial properties, preventing secondary caries 3. Low cytotoxicity and color close to natural teeth	1. High optimal loading (8 wt.%), which may affect handling and material homogeneity 2. Long-term fluoride release characteristics and rechargeability are not clearly defined
		BaO and MgO [34]	1. Significantly increases compressive strength 2. Enhances properties by modifying the glass composition itself, avoiding issues associated with introducing secondary phases	1. Excessively long setting time(~16 min), resulting in poor clinical handling 2. Research is still in its early stages, lacking crucial data on long-term durability, fluoride release, and adhesion
Powders	Fiber	Glass short fibers (CPSA) [38]	1. Remarkable strengthening effect 2. High reactivity with the mixing liquid/cement matrix, leading to strong bonding 3. High tensile strength of the fiber itself 4. Possesses excellent biocompatibility and bone conduction	1. High fiber content (60 mass%) required for optimal performance 2. Setting time prolonged with increasing fiber content 3. Complex preparation process for the fibers 4. Limited improvement in resilience
		Basalt Fiber [39]	1. Significantly improved mechanical properties 2. Environmentally friendly production, more energy-saving than glass fibers 3. Excellent chemical stability	1. Weak interaction between fibers and cement matrix, leading to decreased properties after water aging 2. Natural brown color limits esthetic applications 3. Handling ability impaired at high loading (>9 wt.%) 4. Fibers mainly showed pull-out and debonding, indicating weak interfacial adhesion
		Cellulose fibers [40,41,42,43]	1. Significantly enhance mechanical performance 2. Good biocompatibility and safety potential 3. Good physical compatibility with the GIC matrix 4. Economy and sustainability	1. Operational challenges 2. The interfacial bonding strength is relatively weak 3. Performance improvements involve trade-offs and an optimal window 4. Negatively affecting esthetic performance
		Flax fiber [36]	1. Changed failure mode from brittle to plastic, increased resilience and strain 2. Significantly increased compressive and flexural strength at appropriate loadings 3. No significant effect on the setting reaction kinetics 4. Eliminates health and safety concerns during handling	1. Reduction in compressive and flexural modulus 2. Lack of chemical bonding between fibers and GIC matrix, likely resulting in a weak interface 3. Surface modification (e.g., silane treatment) suggested for future improvement 4. High fiber content (25 wt.%) needed for maximal compressive strength
		Hemp fiber [37]	1. Significant enhancement in flexural strength at low loading (1 wt.%) 2. Biocompatible, easy handling and mixing 3. Cost-effective, offers high yields at low costs 4. Sustainable and eco-friendly, low carbon footprint	1. Narrow optimal loading window, performance declines or roughness increases at higher concentrations (3%, 5%) 2. Fiber-matrix interaction is limited, untreated fibers may have weak chemical bonding with the GIC matrix 3. Lack of knowledge about performance in the oral environment
Powders	Hydroxyapatite	HA [52,53,54]	1. Excellent biocompatibility and ion-releasing properties; high chemical affinity to tooth structure 2. Accelerates early strength development; high strength achievable as early as 15 min 3. Does not interfere with the inherent fluoride release or dentin bonding ability of GIC	1. Reinforcement effect is highly dependent on particle size and morphology 2. Primarily improves toughness; the absolute strength increase may be limited
	Glass hybrids	ChemFil Rock [64]	High overall mechanical strength	Poor surface hardness and wear resistance
		EQUIA Forte Fil [65,66]	1. Enhancement of mechanical performance and durability 2. Effective fluoride release 3. Indicated for stress-bearing restorations	1. Insufficient toughness 2. Inadequate esthetic properties
	Other materials	Carboxymethyl chitosan [67]	1. Significantly enhance mechanical strength 2. Excellent biocompatibility and cell proliferation activity 3. Improving acid erosion resistance stability 4. Optimize operational performance, Reduce the setting time	1. Costs may be relatively high 2. Long-term performance data requires further refinement
		Reduced graphene oxide [70]	1. Significantly increase hardness 2. Effectively inhibiting crack propagation and enhancing material toughness	1. Dark color, severely restricts esthetic appeal 2. Limited improvement in strength 3. To avoid excessive color intensity, its maximum effective dosage is limited to a very low level (0.1 wt.%)
		Fluorinated graphene [71]	1. Outstanding comprehensive enhancement 2. Markedly improves wear resistance 3. Possesses excellent antibacterial properties against cariogenic bacteria 4. Bright white color does not compromise the esthetics of the restoration	1. Has an optimal loading window; excess loading (>2 wt.%) leads to agglomeration and property degradation 2. Synthesis and dispersion processes are relatively complex 3. The long-term fluoride release profile of the GIC may be altered
		Multi-walled carbon nanotubes [76]	1. Effectively improves compressive strength, hardness, and wear resistance 2. Nano-scale reinforcement; effective at low loadings	1. Dark black color severely compromises esthetics, limiting use to posterior teeth or non-visible areas 2. Similarly faces dispersion challenges; excessive loading (>1 wt.%) may reduce strength 3. Biocompatibility and long-term safety require further extensive study
Liquid	Additives	Collagen [80]	1. Significantly promoted survival of gingival fibroblasts, biointegration 2. Enhanced compressive strength	Higher concentrations of collagen compromised their mechanical property
		Cellulose [81,82,83]	1. Significantly improved the mechanical properties 2. From renewable and abundant sources 3. Significantly increased the fluoride release	1. Risk of aggregation 2. No significant improvement observed in some strength
		Chitosan [84]	1. Catalyzed the fluoride release 2. Significant increase in the flexural resistance 3. High biocompatibility and hydrophilicity	1. CH contents larger than 0.022 wt.% led to poor performance 2. Addition of CH under acid conditions is mandatory
		Carboxymethyl chitosan [85]	1. Enhance the fracture properties 2. Decreased the solubility of GIC 3. Good immersion aging-resistance	1. Excessive introduction of CMCS would impair the mechanical performance 2. FS decreased significantly after 1 week of aging time
	Novel copolymers	Amino acid derivatives [86,87,88]	1. Significant improvement in mechanical strength 2. Reduced brittleness 3. Potential biocompatibility 4. Tunable Polymer Structure	1. Potential decline in handling properties 2. Molecular weight, monomer ratios, and powder/liquid ratio require strict optimization 3. The synthesis and purification steps are more involved 4. Long-term stability not fully validated
		N-vinyl lactam [89,90,91,92,93]	1. Significantly improve mechanical performance 2. Improve operation and solidification performance 3. Excellent biocompatibility	1. Potential increase in post-curing water absorption 2. Long-term stability not fully validated 3. Aggregation control is difficult 4. The optimal mole ratio is specific to a particular glass powder
		Alkyl-Enoic acids [94,95,96]	1. Significantly improved mechanical properties 2. Good water aging resistance and fluoride release 3. Tunable and with design flexibility	1. The enhancement effects of different alkyl alkenoic acids vary significantly 2. The polymerization process and molecular weight control require high precision 3. Long-term stability remain to be observed 4. It may lead to increased viscosity during clinical mixing and a shortened working time

## Data Availability

No new data were created or analyzed in this study. Data sharing is not applicable to this article.
